# Use of Eggshells as Bone Grafts around Commercially Pure Titanium Implant Screws Coated with Nano Calcium Sulfate

**DOI:** 10.1155/2022/8722283

**Published:** 2022-08-10

**Authors:** Dher Riyadh Kadhim, Thekra Ismael Hamad, Abdalbseet A. Fatalla

**Affiliations:** ^1^Ministry of Health, Basra, Iraq; ^2^Department of Prosthodontics, College of Dentistry, University of Baghdad, Baghdad, Iraq

## Abstract

**Background:**

Implant insertion in regions with poor bone quantity, such as the posterior maxilla, is potentially associated with an increased rate of implant failure. Calcium sulfate can be used as the coating material for commercially pure titanium (CpTi) and as the bone graft material around implants when bound to eggshell powder to enhance the bone quality and quantity of bone defect regions. This study performed a torque removal test to evaluate the effectiveness of eggshell powder as a bone substitute for filling bone defects around CpTi-coated implants coated with nanocrystalline calcium sulfate.

**Materials and Methods:**

Eighty screw implant designs were used in the tibiae of 20 white New Zealand rabbits. A total of uncoated 20 screws constituted the control group, and the remaining 60 screws coated with nano calcium sulfate nanoparticles were used as the experimental groups as follows: 20 screws coated with nano calcium sulfate were used alone in the tibiae without gaps around them, 20 screws coated with nano calcium sulfate were used with the gaps made around them and filled with eggshell powder as the bone graft material, and 20 screws coated with nano calcium sulfate were used with the gaps made around them left unfilled.

**Results:**

After 2 to 6 weeks of healing, a significant improvement in bone regeneration and an increase in torque removal values were observed when the bone defect around the CpTi implant coated with nano calcium sulfate was filled with eggshell powder as the bone substitute.

**Conclusions:**

Nano calcium sulfate particles applied through the dip-coating method can successfully work as the coating material of CpTi implants. These particles work in synergy with eggshell powder to act as the bone graft around the implants.

## 1. Introduction

Titanium dental implants are functional structures with the main aim of correcting edentulism [1]. In the last few decades, several coating procedures and materials for changing surfaces of dental implants have been introduced to achieve the best biological properties and biocompatibility. These modifications intend to enhance and hasten osseointegration, which is regarded as a keystone of the success of dental implants, particularly in patients with defective bone healing [2–5].

Many coating techniques for enhancing the potential of implants to engage with healthy bones have emerged. The main principle of coating is to insert a thin bone substitute material that attaches to the implant and bone while supplementing the bone [6–8]. Dip coating is regarded as one of the best procedures for obtaining a thin biocompatible ceramic film on titanium dental implants for enhancing osseointegration. This method is more widely used than other procedures, e.g., spin coating, because it is highly controllable and widely relevant to the sol-gel dip-coating technique [9–11].

Calcium sulfate is a synthetic ceramic bone substitute material with osteoconductive abilities. It is a bioinert material that is readily resolved when inserted inside the human body, and a fibrovascular tissue appears in weeks, leading to neovascularization and bone formation in an area without considerable inflammatory reaction [12]. Calcium sulfate may facilitate the healing of human bone defects caused by surgical implantation [13].

Nano calcium sulfate has been successfully used to coat commercially pure titanium (CpTi) implants through the dip-coating technique by using polyvinyl alcohol (PVA) and polyvinylpyrrolidone (PVP) as binding agents and sintering at 550°C for 1 h. This method yields a uniform coated layer, minimal noticeable cracks with a lowest coating material loss, and the highest roughness value [14].

Eggshell is considered a good bone substitute in peri-implant defects and interposition grafts because of its biocompatibility and ability to bond with the bony site [15–17]. It is composed of CaCO_3_ (almost 93.7%) [8] and has been shown to be a good bone graft material, especially in maxillofacial surgery procedures, due to its mineral composition and good mechanical properties.

The thicknesses of eggshells range from 200 *μ*m to 400 *μ*m. The main components of eggshells are minerals (96.1%), proteins (3.3%), and water (1.6%) [8].

The calcified eggshell consists of an organic ground substance that accounts for almost 3% of its total weight. Moreover, calcified eggshell contains proteoglycans and proteins, such as ovalbumin, ovocleidin-116, lysozyme, povocalyxin-32, ovocleidin-17, and osteopontin, among which some can change the rate of calcite precipitation and the crystal morphology of eggshells [18].

The eggshell powder has a positive effect on bone metabolism and is a good candidate for bone grafting due to its safety and biodegradability [19].

CaCO_3_ derived from eggshells has been described to be an effective osteoconductive and biocompatible biomaterial in many experimental animal studies [20].

This study performed the torque removal test to estimate and compare the effect of eggshells as a bone substitute on bone defects around CpTi implants coated mechanically with nanocrystalline calcium sulfate.

## 2. Materials and Methods

### 2.1. Implant Preparation

Eighty screws were prepared by using a lathe-cutting machine with a titanium carbide cutting head. Each screw was 8 mm in length (the threaded part was 6 mm and the nonthreaded part was 2 mm) and 3 mm in diameter. A slit with a width of 1 mm and a depth of 1.5 mm for fitting a screwdriver and a torque meter during insertion and removal was located centrally in the screw head.

The screws were cleaned first by using a solution consisting of a mixture of acids (30% HNO_3_, 10% hydrofluoric acid, and 60% H_2_O) and distilled water to remove surface contamination and obtain a clean and uniform surface [21]. Then, an ultrasonic bath with absolute ethanol (≥99.8%) was used to remove any remaining debris, contaminants, and acids from all screws. Ultrasonic cleaning in ethanol was performed for up to 15 min, and then, the screws were cleaned in a distilled water bath for 10 min. Finally, all screws were dried at room temperature [4].

For implant coating, the proximal unthreaded portion of the implant must be sufficiently smooth to minimize the adherence of bacteria that can cause adverse mucosal tissue reactions as a result of crestal bone loss [22]. Only the 6 mm threaded part of the screw, which would be completely introduced into the bone tissue of the rabbit tibia, must be coated for standardization and to avoid the effects of the coating on the unthreaded area from interfering during histological and torque meter analyses. However, the removal of the coating layer from the unthreaded area of the screw can affect the remaining coated layer on the threaded part through cracking or partial loss of the coating layer. For these reasons, the unthreaded part of each screw was wrapped by using a tight, heat-shrinking plastic wire sleeve before coating [23].

### 2.2. Dip-Coating Technique

#### 2.2.1. Preparation of Nano Calcium Sulfate Coating Solution

The nano calcium sulfate (commercially purchased from Orthogene Corp., USA) coating solution was prepared as follows:

1. A total of 10 g of nano calcium sulfate was added to 50 ml of NaCl in a glass container. The mixture was heated at 45°C on a hot plate stirrer for half an hour to obtain a homogenous solution in accordance with the manufacturer's instructions.
2. A total of 0.01 g of P_2_O_5_ was added to the previous solution.
3. A second solution was prepared through the addition of 0.5 g of PVA to the solution of 10 g of nano calcium sulfate in 50 ml of NaCl. The temperature was maintained at approximately 45°C. The mixture was stirred for half an hour to obtain a homogenous solution.

The third solution was prepared through the same procedure used to prepare the second solution except that 0.5 g of PVP was added to 0.5 g of PVA.

Screws (*n* = 60) were used for the dip-coating technique. By using a coating device, each screw was dipped into the prepared nano calcium sulfate solution for 60 s four times and then withdrawn with a well-defined withdrawal speed.

After dipping, the samples were air-dried for 24 h. Then, the sleeves were removed from the screws. A tube furnace was used to sinter the coated screws for coating densification. Heat treatment was conducted at 550°C for 1 h.

### 2.3. Sterilization

Each screw was kept in a single airtight sheet before sterilization. The titanium screws were sterilized through gamma irradiation at the dose of 2 megarads by using a Gamma Cell 220 apparatus with a CO_60_ source. This dose is required for medical and surgical equipment sterilization. Gamma irradiation sterilization was done in the radiation department of Al-Amal Hospital in accordance with the Atomic Energy of Canada Limited (1984). The radiation used had an energy of approximately 1.25 MeV at the dose rate of 90.4 rad/min. The distance between the screws and the radiation source was 65 cm, and the exposure time was 60 min.

### 2.4. Preparation of Eggshell Powder as the Bone Substitute Material

Eggs were purchased from a local market. The eggs were rinsed with distilled water, then broken and emptied of their whites and yolks. Their membranes were peeled off manually, and the eggshells were ground into a powder with a porcelain pestle and mortar and kept in a desiccator to avoid humidity. The eggshell particles were sterilized through autoclaving at 136°C for 18 min [17]. Then, the powders with the largest and smallest particle sizes were analyzed through Fourier transform infrared spectroscopy and phase identification X-ray diffraction.

Nano calcium sulfate was subjected to heat treatment for densification by using a carbonated furnace (tube furnace) in the presence of an inert gas (argon) to avoid the oxidation of the titanium disc. The discs coated with nano calcium sulfate were sintered at 200°C, 400°C, 550°C, and 600°C for 2 h. The most appropriate heat treatment was 550°C for 1 h.

### 2.5. Experimental Animal and Implantation Procedure

Twenty adult male New Zealand rabbits (weighing 2–2.5 kg, aged 10–12 months) were used in this study. The rabbits were all kept in special cages (40 cm × 60 cm × 70 cm) and received a single dose (10 mg/kg B.W.) of ivermectin as a prophylactic measure against most internal and external parasites [24].

Initial holes were drilled into the rabbit tibia by using a handpiece (QD, England) with intermittent and continuous cooling with irrigated normal saline. An approximately 6 mm mark was made on the bur to determine the depth of the hole of the screw.

Two holes with a diameter of 2.5 mm and a distance of 1 cm were prepared. The upper left hole was filled with a sterilized uncoated screw. The lower left hole was filled with a sterilized screw coated with nano calcium sulfate. The sterilized uncoated screw was inserted by using a screwdriver until the screw thread was completely introduced into its bed.

A professional torque meter (TQ 8800, Mrclab, China) was used to check the final introduction of the screw into the bone tissue and to measure the insertion torque force, which ranged from 0.9 N/cm to 1 N/cm. The same technique was repeated on the right tibia. Then, a marker was used to create a 2 mm mark on the bur that was reinserted in the hole such that the end of the marker was in level with the surface hole to create a 2 mm space or gap around the coronal part of the screw.

A screw coated with nano calcium sulfate was inserted into the upper right hole, and the gap was filled with eggshell powder two times by using an amalgam carrier. The same method was used to treat the lower right hole in the control group (without filling the gap with eggshell powder).

Eight implants from all group tests were taken at each healing interval (2 and 6 weeks) for the determination of biological function by using a professional removal torque tester (TQ 8800, Mrclab, China). Radiographic images (Eco ray, Korea) were taken at 2 to 6 weeks with light anesthesia to control the rabbits. Images were taken in the mediolateral view and the anterior-posterior view with the following exposure factors: Kv = 47–54, mAs = 2.20–4, and focus film distance = 30 cm. The X-ray films were processed in a darkroom by using an automatic processor.

## 3. Results

### 3.1. FTIR Analysis

**Figure 1:**
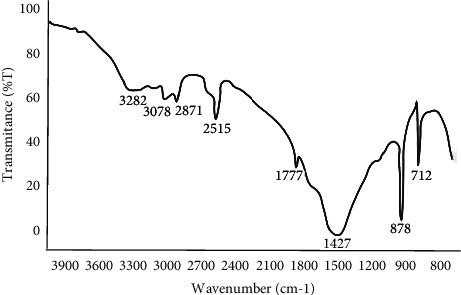
FTIR analysis for the eggshell particle of less than 50 *μ*m.

**Figure 2:**
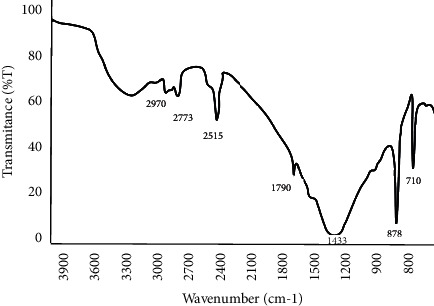
FTIR analysis for the eggshell powder of a particle size of 150 *μ*m.

The spectra of the eggshell powders with particle sizes of less than 50 *μ*m and 150 *μ*m were obtained. The spectra of the eggshell powder with a particle size of less than 50 *μ*m showed a peak at 1433 that was related to the carbonate material inside the matrix of the eggshell [25]. The peaks at 710 and 876 were related to the sequential deformation from the inner side of the plane and deformation from the outer side of the plane of CaCO_3_. The remaining peaks (3288, 2979, and 2873) were related to amines and amides [26]. The eggshell powder with the particle size of 150 *μ*m had the same, but slightly larger, peak variation as the other eggshell powder samples (Figures 1 and 2).

The bands 2971–3078 cm^−1^ represent a C-H vibration that indicates the existence of an organic layer, built from amino acids in the eggshells. While the band 1777 cm^−1^ corresponds to C=O and carbonyl group stretching (that is, an amide group).

The existence of calcium carbonate CaCO_3_ refers to observable peaks at 712 and 878 cm^−1^ which are corresponding to inner plane deformation and out-plane deformation, respectively.

### 3.2. Phase Identification

**Figure 3:**
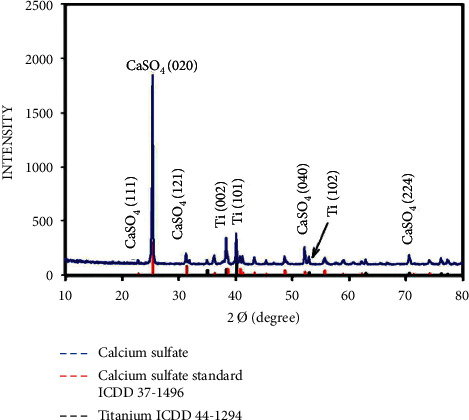
X-ray diffraction pattern of Cp Ti coated with nano calcium sulfate and PVA as a binding agent.

The XRD pattern of CpTi coated with nano calcium sulfate powder in the presence of PVA (Figure 3) showed identical matches with ICDD 37–1496 for calcium sulfate peaks at (111), (020), (121), (040), and (224) and identical matches with ICDD 44–1294 for titanium peaks at (002), (101), and (102). PVA was not found because it evaporated after heat treatment.

**Figure 4:**
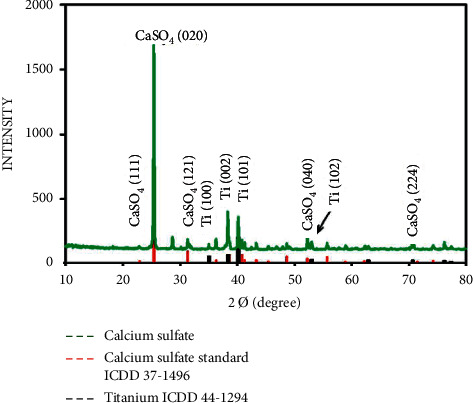
X-ray diffraction pattern of Cp Ti sample coated with nano calcium sulfate and (PVA, PVP) as a binding agent.

The XRD pattern of titanium coated with nano calcium sulfate powder in the presence of PVA and PVP (Figure 4) showed identical matches with ICDD 37–1496 for calcium sulfate peaks at (111), (020), (121), (040), and (224) and identical matches with ICDD 44–1294 for titanium peaks at (100), (002), (101), and (102). PVA and PVP did not appear because they evaporated after heat treatment.

**Figure 5:**
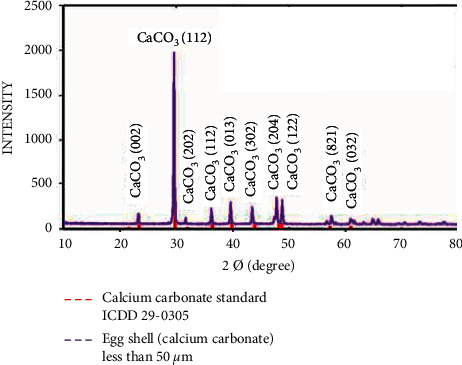
X-ray diffraction patterns for the eggshell with a particle size of less than 50 *μ*m.

The XRD pattern of the eggshell powder with a particle size of less than 50 *μ*m (Figure 5) showed an identical match with ICDD 29–0305 in terms of the peaks at (002), (112), (202), (112), (013), and (032) and slight shifts in peaks (302), (204), and (821) because the eggshell powder required temperatures that were higher than those used for sterilization to reach identical matches and perfect crystallization.

**Figure 6:**
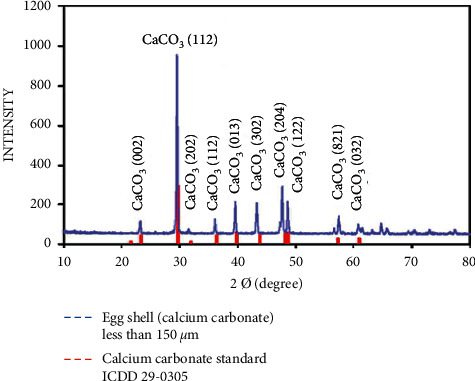
X-ray diffraction pattern for the eggshell particle size of 150 *μ*m.

Peaks (002), (112), (202), (112), (013), (032), and (821) in the XRD pattern of the eggshell powder with a particle size of 150 *μ*m (Figure 6) showed identical matches with those in ICDD 29-0305. In contrast to that observed in Figure 5, a slight shift in peaks (302) and (204) was observed due to the larger particle size of the powder. Thus, the amount of calcium carbonate detected by XRD increased.

### 3.3. Radiographical Examination

Radiographic evaluation was performed to show the contact between the bone and implant. The radiographical evaluation found no radiolucent regions or any abnormality activity from the bone to the implant at the end of the 2 to6 week healing periods. Given the difficulty experienced by clinicians in diagnosing radiographic bone loss at 0.1 mm resolution, the absence of radiolucency in the implantation site is not a sign of osseointegration.

### 3.4. Mechanical Test

Implant resistance to torque removal has been related to the level of bone-implant contact by many reports that connected the alteration in biomechanical features during regeneration with the dynamics of bone-interface healing [27].

In this study, the removal torque was considered an index of the presence of osseointegration and a representation of the mechanical properties of the bone-implant interface.

The removal torque is the torsional force that is required for removing an implant fixture and is measured in N/cm by using a digital torque meter. This measurement technique is primarily used to determine interfacial shear properties, and its results may be related to implant geometry and topography [20, 28].

Table 1 shows the descriptive statistics for the mean removal torques of the CpTi screws coated with nano calcium sulfate at 2 to 6 weeks after implantation (Table 1).

After 2 weeks, the mean value of the torque needed to remove the implants in group *C* increased to 13.000 N/cm.

In addition, after 6 weeks, the mean value of the torque needed to remove the implants in group *C* increased to 17.375 N/cm. This value was higher than the values obtained for the remaining groups.

**Figure 7:**
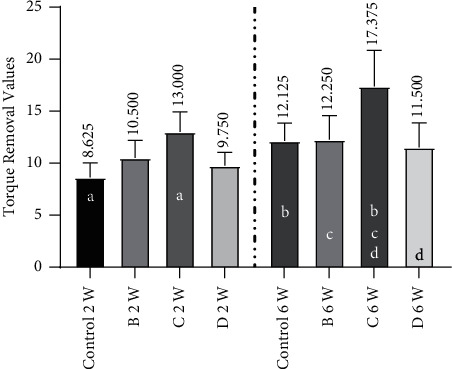
Bar chart with a mean and standard deviation for the torque removal test after 2 to 6 weeks of healing. Groups with a similar lower case letter considered significance at *p* < 0.05 using Wilcoxon ranked test.

The *t* test was performed to analyze the equality of means between the two healing intervals for all groups. A significant difference at *p* ≤ 0.01 was shown by the control group *A* and group *B*. Significant differences (*p* < 0.05) were shown by groups *C* and *D* (Figure 7).

## 4. Discussion

### 4.1. FTIR Analysis of Eggshell Powder

The FTIR result of eggshell particles confirmed that calcium carbonate was the main component. The peaks at 710, 876, 1433, 1799, 2515, and 2873 in the spectra of the eggshell powder with a particle size of 50 *μ*m indicated that CaCO_3_ was the major component. The absorption peaks at 2873, 2979, and 1799 were related to CO_3_^−2^; the strongest peak at 1433 corresponded to C–O, and the peak at 3288 was related to the O–H group in accordance with previous findings [21]. The eggshell powder with a particle size of 150 µm showed the same peak variations as the eggshell powder with a particle size of 50 *μ*m.

### 4.2. In Vivo Experiments

#### 4.2.1. Experimental Animals

Adult male New Zealand white rabbits were chosen because of their rapid osseointegration rate, easy control, availability, and lower costs than other animals. In addition, these strains have a nonaggressive nature with a low incidence of health problems. Furthermore, many implant reports have used the rabbit tibia as a successful implantation site [29, 30].

In this study, the ages of the rabbits approximately ranged from 10 months to 12 months because, at these ages, the proximal tibia of the rabbits physiologically resembles that of adult humans [31].

The tibial area of the rabbits was chosen to simulate the clinical situation given that the dimensions of this bone almost resemble those of the human alveolar space. The tibia is considered a suitable implant location site for various reasons. The surgical part has a low level of morbidity because the medial proximal tibia can be approached easily for the placement of the implant and the cancellous bone acts as a cushion to avoid the splinting of the cortical bone during surgery. These findings are in agreement with the results of Mapara et al. [32] and Ibrahim et al. [33].

Dupoirieux et al. [8] reported that eggshell powders with a particle size of 50 *μ*m are associated with faster bone healing than those with larger particles. Therefore, in the present study, eggshell powder with a particle size of approximately 50 *μ*m was chosen as the bone graft material.

#### 4.2.2. Clinical Tests

The primary and secondary stabilities of all implants were evaluated on the basis of mobility detection by using an instrument with a blunt end. This test revealed whether the implant was stable or not after implant insertion and at the end of the healing interval. This test was based on implant resistance to removal by manual force and was done without the help of a torque meter. These evaluations were conducted to determine if the CpTi screws were successfully coated with nano calcium sulfate. The results obtained in this work were similar to the findings of Mistry et al. [34] and Smeets et al. [35], who demonstrated that the achievement and preservation of implant stability are requirements for successful implants.

In this study, the uncoated and coated implants were exposed to gamma irradiation. Irradiation sterilization, rather than autoclaving, was performed to remove surface contaminants to avoid the accumulation of salts that may be present in autoclave steam [36]. In 2009, Silindir and Ozer [37] suggested that in contrast to various sterilization methods (autoclaving and exposure to UV light), the sterilization of implants by gamma irradiation produced an obvious surface as confirmed by the increase in the thickness of the oxide layer and the reduction in the wetting angles. Gamma sterilization has no negative effect on the surface composition of titanium discs [28].

The absence of signs of any severe infection in the implantation area after each interval indicated that the material was tolerated. Excellent conditions for implantation involve implant sterilization, aseptic surgical procedures, and instrument autoclaving. The precise use of sharp drills during surgery and the application of cooling and discontinuous pressure to prevent the overheating of bone and the distribution of necrotic bone in the implant bed were other factors to be considered for the establishment of osseointegration.

#### 4.2.3. Mechanical Test

Implant resistance to torque removal has been related to the level of bone-implant contact by many reports that connected alterations in biomechanical features during regeneration to the dynamics of bone-interface healing [38].

Torque is the movement or twisting applied by the load at the span of the body that is identical to the load multiplied by the horizontal span between the center of rotation and the line of action at which it is applied [39]. In this study, removal torque was considered an indicator of osseointegration and a mechanical property of the bone-implant interface.

Removal torque is the torsional force essential for removing an implant fixture. It is measured in N/cm by using a digital torque meter. This technique is primarily used to measure interfacial shear properties, and its results may be related to implant geometry and topography [23, 24].

In this study, the removal torque values were found to significantly increase with time in groups *B* and *C*, likely because the resulting bone formation in the implant-bone-coating interface was due to bone remodeling around the implant during healing that consequently improved mechanical capacity. Simultaneous bone graft and implant insertion shortened the healing interval without increasing the risk of complications or decreasing the success rates [25]. This result can be attributed to the bioactive mechanism of calcium sulfate bone formation because calcium sulfate dissolved into calcium and sulfate ions when it was implanted inside the bone defect and came into contact with the body fluid. In addition, the phosphorus ions that were released from the body fluid were combined with calcium ions, thus forming calcium phosphate, which was considered a self-forming biological appetite on the surface of calcium sulfate. Calcium sulfate was slowly dissolved in body fluid, leading to the deposition of carbonate apatite, which was stable under the same environment and then transformed into bioactive elements that induced bone formation. This mechanism was consistent with previously reported mechanisms [40] and accounted for the highly significantly different results of group *B* at subsequent intervals.

In addition, the combination of calcium sulfate with other graft materials led to increased bone production and improved handling and molecule graft containment. Compared with the use of allograft material alone, the use of the eggshell bone graft material resulted in higher bone production at the defect site. Thus, in agreement with the results of Al Ruhaimi [41] and Uraz et al. [42], bone quantity regeneration was higher when the eggshell bone graft material was applied than when the defects were left unfilled due to the increase in the quantity of mineralized new bone as a result of biodegradation, which changed the grafted particles in new bone. This phenomenon accounted for the significant difference shown by group *C* in the paired *t* test (Figure 7), which revealed that the bonding intensity of the implant interface had increased. Furthermore, a previous work [34] reported that the mechanical test cannot be used to verify osseointegration. Therefore, in this study, histological analysis was performed to verify osseointegration in each group.

## 5. Conclusions

The dip-coating technique can successfully coat CpTi with nano calcium sulfate in the presence of PVA and PVP as the binding agent as proven by the presence of the homogenous polycrystalline structure of calcium sulfate nano particles on the titanium surface and the increase in surface roughness. The experimental group (Eggshell of 50 μm particle size used as a bone graft material in addition to coating titanium implant screw with nano calcium sulfate-material) shows the highest mean value for removal torque in 2 and 6 weeks time intervals of implantation

## Figures and Tables

**Table 1:** Descriptive statistical analysis for the torque removal test after 2 to 6 weeks of the healing period.

	*N*	Mean N. cm	Std. deviation	Std. error	95% confidence interval for mean		Minimum	Maximum
					Lower bound	Upper bound		
Control 2w	8	8.625	1.408	0.498	7.448	9.802	7	11
B 2w	8	10.500	1.690	0.598	9.087	11.913	8	13
C 2w	8	13.000	1.927	0.681	11.389	14.611	10	16
D 2w	8	9.750	1.282	0.453	8.678	10.822	8	12
Control 6 w	8	12.125	1.727	0.611	10.681	13.569	10	15
B 6w	8	12.250	2.315	0.818	10.315	14.185	10	17
C 6w	8	17.375	3.462	1.224	14.481	20.269	12	23
D 6w	8	11.500	2.390	0.845	9.502	13.498	9	16

## Data Availability

The data used to support the findings of this study are available from the corresponding author upon request.
